# DNA Damaging Agents Induce RNA Structural and Transcriptional Changes for Genes Associated with Redox Homeostasis in *Arabidopsis thaliana*

**DOI:** 10.3390/plants14050780

**Published:** 2025-03-04

**Authors:** Ping Li, Jiong-Yi Li, Yu-Jiao Ma, Xiao-Wei Wang, Jian-Ping Chen, Yi-Yuan Li

**Affiliations:** 1 State Key Laboratory for Quality and Safety of Agro-Products, Key Laboratory of Biotechnology in Plant Protection of MARA, Zhejiang Key Laboratory of Green Plant Protection, Institute of Plant Virology, Ningbo University, Ningbo 315211, China; 2 Ministry of Agriculture Key Lab of Molecular Biology of Crop Pathogens and Insects, Institute of Insect Sciences, Zhejiang University, Hangzhou 310058, China

**Keywords:** DNA damage, RNA secondary structure, redox homeostasis

## Abstract

Living organisms are constantly exposed to various DNA damaging agents. While the mechanisms of DNA damage and DNA repair are well understood, the impact of these agents on RNA secondary structure and subsequent function remains elusive. In this study, we explore the effects of DNA damaging reagent methyl methanesulfonate (MMS) on arabidopsis gene expression and RNA secondary structure using the dimethyl sulfate (DMS) mutational profiling with sequencing (DMS-MaPseq) method. Our analyses reveal that changes in transcriptional levels and mRNA structure are key factors in response to DNA damaging agents. MMS treatment leads to the up-regulation of arabidopsis *RBOHs* (respiratory burst oxidase homologues) and alteration in the RNA secondary structure of *GSTF9* and *GSTF10*, thereby enhancing mRNA translation efficiency. Redox homeostasis manipulated by RBOHs and GSTFs plays a crucial role in MMS-induced primary root growth inhibition. In conclusion, our findings shed light on the effects of DNA damaging agents on RNA structure and potential mRNA translation, which provide a new insight to understand the mechanism of DNA damage.

## 1. Introduction

Cells are constantly exposed to DNA damage. Such damage may result from exposure to specific chemicals in the environment, UV light, ionizing radiation, and errors in DNA replication and proofreading, which can affect the integrity of the plant genome and adversely influence plant growth. DNA damaging agents, such as methyl methanesulfonate (MMS), hydroxyurea, narciclasine, heavy metal cadmium (Cd^2+^), and cisplatin, can induce inhibition of plant root growth [1,2,3,4,5]. γ-radiation and MMS have also been found to inhibit the growth of arabidopsis seedlings [6]. Therefore, studying how plants respond to DNA damaging reagents is essential.

To maintain genome stability, plants have evolved a sophisticated system to finely regulate the processes associated with DNA repair and damage tolerance [7,8]. Studies in yeast, plants, and mammals have revealed many conserved DNA damage responses. Upon DNA stress, MRN (Meiotic recombination 11 (MRE11)/Radiation sensitive 50 (RAD50)/Nijmegen Breakage Syndrome 1(NBS1)) and Ku70/80 complexes recognize the damaged DNA [9,10,11,12]. Then, SOG1 (SUPPRESSOR OF GAMMA RESPONSE 1) is phosphorylated and thus activated by ATM/ATR (ATM: ataxia telangiectasia-mutated gene, ATR: ataxia telangiectasia and Rad3-related) to initiate a signaling cascade to cope with DNA damage [13,14,15]. ATM specifically responds to DNA double-strand breaks (DSBs), and ATR primarily senses replication stress caused by a persistent block of replication fork progression to keep faithful duplication. So far, DSBs repaired are known to be in two main ways: non-homologous end-joining (NHEJ) and homologous recombination (HR) [16,17]. HR mediates repair using a homologous sequence as a template and is a very high fidelity repair process, and NHEJ joins DNA ends with little or no dependence on DNA sequence homology [18]. DNA damaging agents could induce many kinds of damage types on DNA. Based on the DNA damaging agents origin, DNA damage is broadly classified as endogenous or of exogenous origin. Deamination, alkylation, oxidation of nitrogenous bases, and DNA breaks are DNA damage types of endogenous origin [19]. Dimerisation, formation of bulky adducts in DNA, DNA breaks, alkylation, and tautomeric isomerism of nitrogenous bases are the main DNA damage types of exogenous origin. In addition, DNA alterations in the information content and structure also occur with exposure to DNA damaging agents [20]. Although DNA damaging factor-induced DNA damage type and its repair mechanism are well known, the effects of DNA damaging agents on RNA secondary structure are ambiguous.

Structural perturbations in RNA molecules may play critical roles in a variety of biological processes ranging from ligand sensing to the regulation of translation, polyadenylation, and splicing [21]. Therefore, obtaining direct data on RNA structure in vivo is essential for understanding how RNA structure contributes to RNA function. Several approaches have been developed to interrogate specific structural features of RNA molecules [22,23,24]. Traditional methods such as X-ray crystallography, nuclear magnetic resonance, and cryogenic electron microscopy are time-consuming and limited in predicting RNA structure in vivo. In recent years, methods based on biochemical or chemical probes have been developed [24,25,26]. Certain chemicals, including dimethyl sulfate (DMS), α-ketoaldehydes, 1-ethyl-3-(3-dimethylaminopropyl) carbodiimide (EDC), and selective 2′-hydroxyl acylation analyzed by primer extension (SHAPE) reagents, have been utilized to predict RNA structure in plants due to their ability to react with unpaired residues [27,28,29].

DNA damaging reagent-induced perturbations of RNA secondary structures might play crucial, and yet unknown, biological roles in response to external stimuli. Previous studies showed that MMS treatment induced the expression of genes related to redox regulation and DNA repair [30,31]. In this study, we conducted DMS-MaPseq analysis [32] to examine the influence of the DNA damage reagent methyl methanesulfonate (MMS) on both *Arabidopsis* gene transcription and RNA secondary structure. Our results showed that 4133 genes were significantly up-regulated and 4132 genes were down-regulated following MMS treatment. Notably, among these genes, 6 *RBOHs* (respiratory burst oxidase homologues) were up-regulated, and knockout of RBOHD and RBOHF partially mitigated MMS-induced primary root growth inhibition. Additionally, MMS caused the alternation of RNA secondary structure, consequently influencing gene translation. The maintenance of redox homeostasis mediated by glutathione transferase GSTFs played a pivotal role in MMS-induced root growth inhibition. Our findings elucidate the interrelationships among DNA damage, RNA transcription, RNA secondary structures, and RNA translation, providing a new method to study the ramifications of DNA damage.

## 2. Results

### 2.1. Library Quality Control

**Figure 1:**
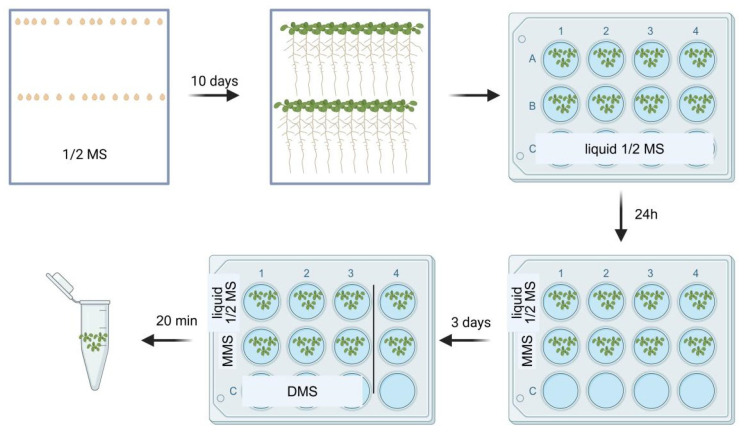
Experimental setup to collect the DMS-MaPseq samples. Ten-day-old Arabidopsis seedlings, which were growing vertically on the Murashige and Skoog (MS) agar medium, were transferred to 24-cell plants, with each cell containing 3 mL liquid 1/2 MS, for one day. They were then treated with 2 mg/mL MMS for 3 days. Following this, the seedlings were immersed into a 2% DMS reaction solution for 20 min and then quenched by addition of β-mercaptoethanol (final concentration 20%). After washing each cell 3 times with 3 mL water, samples were collected. Each cell contained 15 seedlings.

To investigate the impact of MMS treatment on mRNA secondary structure, we collected DMS-MaPseq samples, as depicted in Figure 1. Samples were categorized into four distinct groups: seedlings without DMS treatment (CK, control check), seedlings with DMS treatments (DMS), seedlings with MMS treatment (MMS_CK), and seedlings treated with both MMS and DMS treatment (MD). Overall, we retrieved a range of 11.4 million to 17.3 million clean paired-end reads for each library (Table S1). After being mapped to *Arabidopsis thaliana* TAIR10 cDNA sequences using Bowtie2, groups DMS and MD exhibited a 74.55% to 89.24% mapping rate, while the control groups CK and MMS_CK presented a 96.16% to 97.86% mapping rate (Table S1). The Pearson Correlation Coefficient (PCC) of mRNA abundance demonstrated high consistency across three biological replicates but low between different groups (Figure S1). Analysis for mapping reads of different RNA types in DMS and MD indicated mRNA was the most enriched RNA type in the libraries (Figure S2).

### 2.2. Transcriptional Changes After MMS Treatment

**Figure 2:**
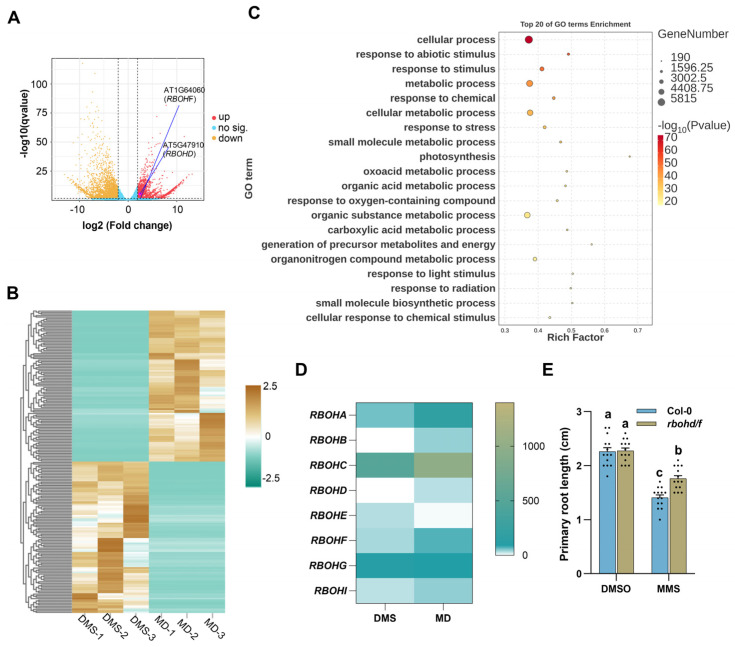
Transcriptome analysis of Arabidopsis seedlings after MMS treatment. (**A**) Volcano plot for differential expression genes (DEGs) with the log2-fold changes analysis between MD and DMS samples. This volcano plot was generated with the log2-fold change cutoff set to ±1 and the q value cutoff set to <0.05. (**B**) Heatmap for DEGs between MD and DMS samples. (**C**) Top 20 enriched GO terms. Gene Ontology (GO) enrichment analysis for biological process of the DEG was performed, and the results were visualized with the OmicShare tool, an online platform for data analysis (https://www.omicshare.com/tools/home/report/goenrich.html). (**D**) Transcripts of RBOHs gene in DMS and MD samples from this study. The data were shown with FPKM values, which were identified by featureCounts software from the Subread package v2.06. (**E**) Arabidopsis *rboh/f* mutants show less hypersensitivity to MMS compared to the wild type Col-0. The seeds were sterilized and subsequently allowed to germinate on half-strength Murashige and Skoog agar plates for 5 days. Following this, the resulting seedlings were transferred to a liquid half-strength Murashige and Skoog medium with or without MMS. Each treatment was applied to 20 seedlings. The lengths of the primary roots were measured 3 days post-MMS treatment. n = 14. Different letters indicate significant differences between treatments (*p* < 0.05).

To uncover *Arabidopsis*’ responses to MMS, we analyzed the differential expression genes (DEGs) between DMS and MD samples using the DMS-MaPseq data. In total, transcripts for 24,598 genes were detected, with 4133 genes being significantly up-regulated and 4132 genes down-regulated after MMS treatment (Figure 2A, Table S2). PCA and heatmap analyses indicated a high level of coordination among the three biological replicates for DMS or MD (Figure 2B and Figure S3). The DEGs were found to be enriched in terms related to cellular processes, metabolism, and responses to stress and abiotic stimuli (Figure 2C). Gene Ontology (Go) analysis of the upregulated and down-regulated genes indicated that genes associated with responses to oxygen levels were up-regulated, while genes related to photosynthesis were downregulated (Figure S4).

### 2.3. Plant NADPH Oxidases Play a Role in Plant Response to MMS

Plant NADPH oxidases, known as Respiratory Burst Oxidase Homologs (RBOHs), play a crucial role in generating reactive oxygen species (ROS). Arabidopsis encodes 10 RBOHs proteins that regulate a diverse array of developmental as well as environmental processes [33]. Previous studies have indicated that MMS can increase the expression of plant *RBOHs* genes, although their specific role in MMS response remains unclear [30]. We also found that, in comparison to DMS treatment, the transcripts of 6 *RBOHs* were significantly upregulated in MD samples (Figure 2D). To investigate the function of RBOHs in MMS response, we focused on RBOHD and RBOHF, known for their role in establishing the protective barrier in plants under biotic and abiotic stress [33]. Results from the primary root growth inhibition experiment revealed that the *rbohd/f* mutant exhibited less sensitivity to MMS (Figure 2E), suggesting that RBOHs play a pivotal role in mediating plant responses to MMS.

### 2.4. RNA Structure Analysis

**Figure 3:**
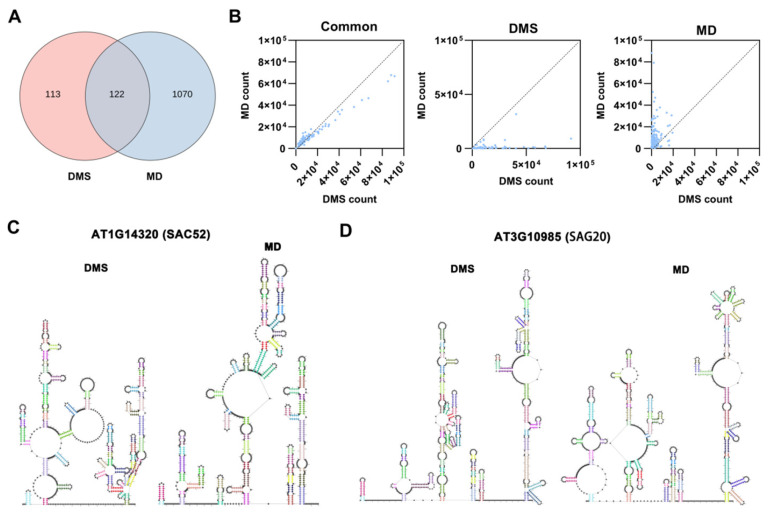
RNA structure analysis for DMS-MaPseq data. (**A**) Venn diagram for the genes with predictable RNA secondary structure in DMS and MD libraries. (**B**) The relationships between count number and common, DMS specific, or MD specific mismatched genes. (**C**,**D**) RNA secondary structure of *SAC52* and *SAG20* predicted by RNArtist.

**Figure 4:**
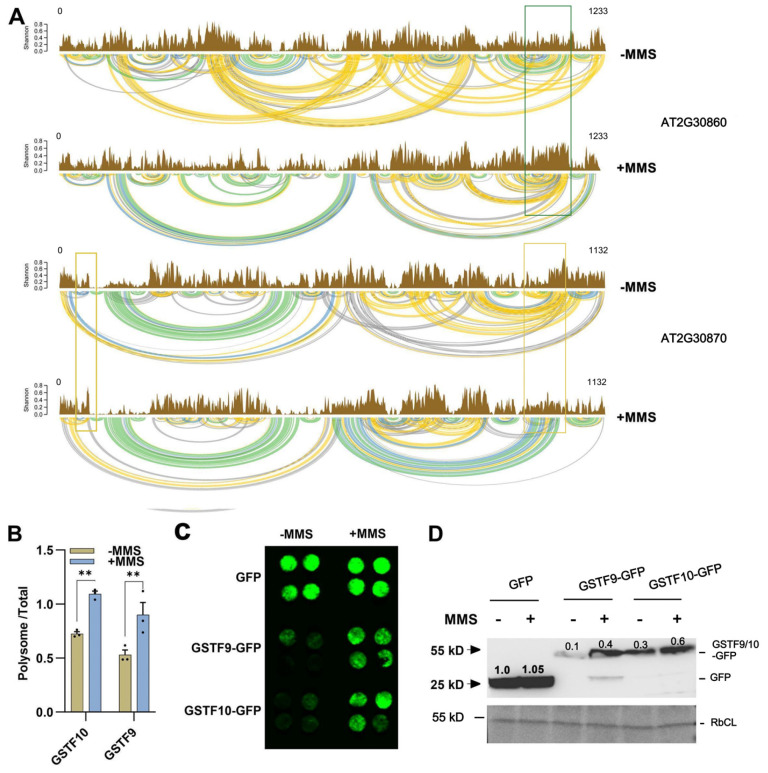
MMS treatment promotes the translation of *GSTF9* and *GSTF10*. (**A**) Base-level Shannon entropy plot and arc plot of base-pairing probabilities. Gene graphical reports (SVG format) were automatically generated by the rf-fold module. Regions with higher Shannon entropies are likely to form alternative structures, while those with low Shannon entropies correspond to regions with well-defined RNA structures or stable secondary structure elements. The lines with different colors in arc plot indicate the base-pairing probabilities. Green: 70–100%; blue: 40–70%; Yellow: 10–40%; gray: 5–10%. The green and yellow rectangular boxes presented the alternative structures near the UTR for *GSTF9* and *GSTF10*. (**B**) MMS treatment increases the translation of *GSTF9* and *GSTF10*. Polysome RNA was extracted after sucrose density gradient centrifugation. The asterisks above the bar indicated significant difference, with ** representing *p* < 0.01. (**C**) GFP signals of GSTF9 and GSTF10 after MMS treatment. (**D**) GFP protein detection of GSTF9 and GSTF10 with immunoblot. Straight lines indicate the expected proteins. The RuBisCo large subunit (RbCL), stained with Ponceau S, served as a loading control.

To validate the RNA structure in vivo, we assessed the DMS-MaPseq data with the workflow depicted in Figure S5. Compared with the reference genome of Arabidopsis thaliana TAIR10, the rf_count mutated alignment rates for CK and MMS were approximately 11%. Conversely, DMS and MD samples exhibited higher rf_count mutated alignment rates ranging from 60% to 85% (Table S3), indicating the potential of our data for predicting RNA structure. Subsequently, we intersected the data from three replicates and compared these results with CK or MMS_CK. In DMS and MD samples, RNA secondary structures for 235 and 1192 genes were predicted, respectively, with 113 genes uniquely enriched in DMS and 1070 in MD (Figure 3A). However, we only obtained one replication for CK and MMS_CK samples, which might affect the final RNA structural prediction results. Considering that mRNA abundance might influence structural prediction results, we investigated the relationship between gene expression level and the potential for RNA structure prediction using the data from the 1305 genes in Figure 4A. A scatter plot revealed that gene expression level significantly influenced RNA structure prediction (Figure 3B). Thus, for samples exhibiting substantial differences in gene expression levels, high sequencing depth should be prioritized.

With DMS-MaPseq data, we predicted the secondary structure for 122 mRNAs in both DMS and MD samples. DNA damaging agent doxorubicin treatment led to a decrease in protein translation due to extensive ubiquitination of ribosomal proteins, resulting in DNA damage [34]. Our data indicated that MMS treatment induced the alternation in RNA secondary structure of *SAC52* (AT1G14320) (Figure 3C), which encodes a highly conserved ribosomal protein L10 in eukaryotes and is implicated in translational regulation. Except for root growth inhibition, we also found that prolonged exposure to MMS could promote leaf senescence (Figure S6), potentially associated with the alternation of the secondary structure of the senescence-associated gene 20 (*SAG20*, AT3G10985) (Figure 3D), which belongs to a group of genes that regulate leaf senescence.

### 2.5. RNA Structures for GSTF9 and GSTF10 Determine Their Translation

Among the 122 genes with predictable RNA structures, we focused on *GSTF9* (AT2G30860) and *GSTF10* (AT2G30870) genes due to their crucial role in regulating plant redox balance. Both genes showed altered secondary structures after MMS treatments (Figure 4A). While MMS treatment had no significant effects on the secondary structure of *GSTF9* mRNA in the 5′-untranslated region and at its start sites, the secondary structure near the 3′-untranslated region became more variable. In contrast, significant structural changes in *GSTF10* mRNA were predominantly observed near both the 5′ and 3′ UTR regions (Figure 4A). Changes in RNA secondary structure, particularly in the UTR region, may influence gene translation efficiency [35,36]. Consequently, we investigated the effects of MMS on the translation of *GSTF9* and *GSTF10*. Total RNA and polysome-bound RNA were extracted and subjected to reverse transcription. Prior to conducting the qRT-PCR experiment, we evaluated the quality of cDNA templates and the amplification efficiency of the primers (Figures S7 and S8). qRT-PCR results showed that MMS treatment enhanced the translation efficiency of *GSTF9* and *GSTF10* (Figure 4B). Additionally, GSTF9 and GSTF10 proteins, fused with GFP, were transiently expressed in the leaves of *Nicotiana benthamiana*. Observations of GFP signals and analyses of protein accumulation were conducted three days after inoculation. The results further supported the conclusion that MMS treatment resulted in increased accumulation of GSTF9 and GSTF10 proteins (Figure 4C,D).

### 2.6. Redox Homeostasis Is Associated with MMS-Induced Response

**Figure 5:**
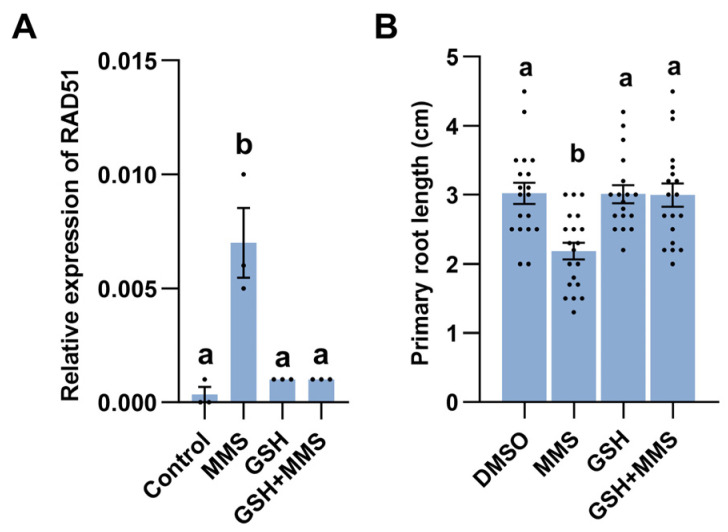
Redox homeostasis plays vital role in MMS-induced response. (**A**) GSH addition alleviates the expression of RAD51 related to DNA damage. (**B**) GSH addition decreases MMS-induced primary root growth inhibition. The seeds were sterilized and subsequently allowed to germinate on half-strength Murashige and Skoog agar plates for 5 days. Following this, the resulting seedlings were transferred to a liquid half-strength Murashige and Skoog medium with or without MMS. Each treatment was applied to 20 seedlings. The lengths of the primary roots were measured 3 days post-MMS treatment. Different letters indicate significant differences between treatments (*p* < 0.05).

Combined with the gene expression data that the majority of GSTF family genes related to redox homeostasis were upregulated after MMS treatment (Figure S9), we hypothesized a connection between DNA damage and redox imbalance. Therefore, we investigated whether the addition of antioxidant molecules reduced glutathione (GSH) and could alleviate MMS-induced DNA damage. qRT-PCR analysis confirmed that exogenous application of GSH could compromise the expression of DNA damage marker gene *RAD51* (Figure 5A). Moreover, the addition of GSH decreased the sensitivity of Arabidopsis seedlings to MMS (Figure 5B). Collectively, these findings suggest that redox homeostasis may play a crucial role in modulating MMS-induced root growth inhibition.

## 3. Discussion

In response to the threat of DNA damage agents, cells exhibit dramatic transcriptional changes. Exposure to 80 Gy X-rays for 3 h has been shown to induce changes in the expression of 2543 genes [37]. A temporal examination of the expression network after exposure to γ-IR revealed an expanded set of DNA damage-responsive genes [38]. Previous studies have also demonstrated that MMS treatment leads to alternation in the expression of mRNA involved in responses to oxidative stress, DNA damage, and the cell cycle [30]. Our finding further indicated that MMS treatment could trigger gene transcriptional remodeling in Arabidopsis. Approximately 4133 and 4132 genes were significantly upregulated and downregulated after MMS treatment, respectively (Figure 2A). The DEGs were enriched in terms related to cellular process, metabolic process, and stress response (Figure 2C). Previous studies have also shown that MMS treatment for 24 h remodels the expression of 3666 genes, the majority of which are associated with plant metabolism. Notably, the DNA repair-related genes RAD51 and RAD54 were upregulated in both studies [31]. However, since the original data are unavailable, we are unable to make a detailed comparison. Additionally, an observed upregulation of genes associated with ROS generation and ROS scavenging, such as *RBOHD*, *RBOHF*, and *GSTFs*, was observed after MMS treatment (Figure 2D and Table S3). A recent study indicates that MMS induces the expression of *RBOHD*, *RBOHF*, *GSH1*, and *GSH2* and promotes the accumulation of ROS [30]. Whereas the role of redox homeostasis in the plant response to MMS has not been verified.

ROS are well recognized as mediators of DNA damage. In *Saccharomyces cerevisiae*, both MMS and UV light-induced DNA damage cause an increase in intracellular ROS [39]. DNA damaging agents such as neocarzinostatin (NCS, a radiomimetic reagent), doxorubicin, or hydroxyurea trigger ROS accumulation in human U2OS and HBL100 cells [40]. In animals, certain DNA damage responses were dependent on NADPH oxidase (NOX)-derived ROS production. Inactivation of NOX4 and NOX5 results in reduced radiation-induced DNA damage [41]. NOX4 also plays a key role in promoting DNA damage in response to PSO in MCF-7 cells [42]. NOX2 is involved in the retention of DNA damage induced by As(III) and UVR in human neonatal epidermal keratinocytes [43]. NOX1 is associated with NCS-induced ROS in U2OS and HBL-100 cells [40]. Although it has been reported that MMS treatment enhances the gene expression of RBOHs and the accumulation of ROS [30], the role of RBOHs in MMS-induced response remains unclear. In this study, we observed that transcripts of 6 RBOHs were up-regulated (Figure 2D). Knockout of RBOHD and RBOHF partially alleviated MMS-induced primary root growth inhibition (Figure 2E), suggesting that RBOHs mediate the plant response to MMS-induced DNA damage.

RNA secondary structure is a pivotal factor in determining RNA function and influences multiple facets of RNA biology, including stability, processing, translation, localization, and interaction with other molecules [44]. Thus, a comprehensive understanding of RNA secondary structure is indispensable for deciphering the intricate networks of gene regulation. While the effects of gene expression, RNA modification, and mRNA decay on gene function have been extensively explored [45,46,47], the impact of RNA structure on gene function remains largely uncharted. Despite the utilization of various methods for RNA structure prediction, certain questions persist, such as the effects of external stimuli on gene structure and the correlation between gene structure and translation. The present study demonstrated that MMS treatment can induce the alternation of RNA secondary structure (Figure 3A). However, it is worth noting that the number of genes with predictable RNA structure may be influenced by gene expression level and sequencing depth. RNA exhibiting higher expression levels tend to have more easily identifiable secondary structures (Figure 3B). Through the analysis of RNA structure, we observed the changes in the mRNA structure of *GSTF9* and *GSTF10* genes, leading to enhanced gene translation and protein accumulation (Figure 4).

To counteract the detrimental effects of ROS, plants deploy antioxidative mechanisms. One crucial detoxification system in plants is the ‘ascorbate–glutathione cycle’. In this cycle, ascorbate peroxidase (APX) utilizes ascorbic acid and oxidizes it to monodehydro ascorbate (MDAR). However, glutathione reductase (GR) converts oxidized glutathione (GSSG) to reduced glutathione (GSH) [48]. The enzyme family glutathione transferase (GST) participates in the recycling of antioxidants. Arabidopsis genome contains 55 GST coding genes, categorized into 8 classes: GSTU, GSTF, GSTL, GSTT, GSTZ, DHAR (dehydroascorbate reductase), TCHQD (tetrachloro hydroquinone dehalogenase), and EF1Bγ (elongation factor 1B, hemerythrin, and Iota). Among them, 28 genes belong to the GSTU class, and 13 genes belong to the GSTF class [49]. GSTs are involved in response to the abiotic stress, including drought, salt, heavy metal stress, and so on [50,51,52]. MMS treatment led to higher expression of genes in the GST family, suggesting the ascorbate-glutathione cycle may be associated with the MMS response. The addition of GSH alleviated MMS-induced RAD51 gene expression and primary root growth inhibition (Figure 5). Therefore, maintaining plant redox homeostasis is crucial for responding to DNA-damaging agents.

**Figure 6:**
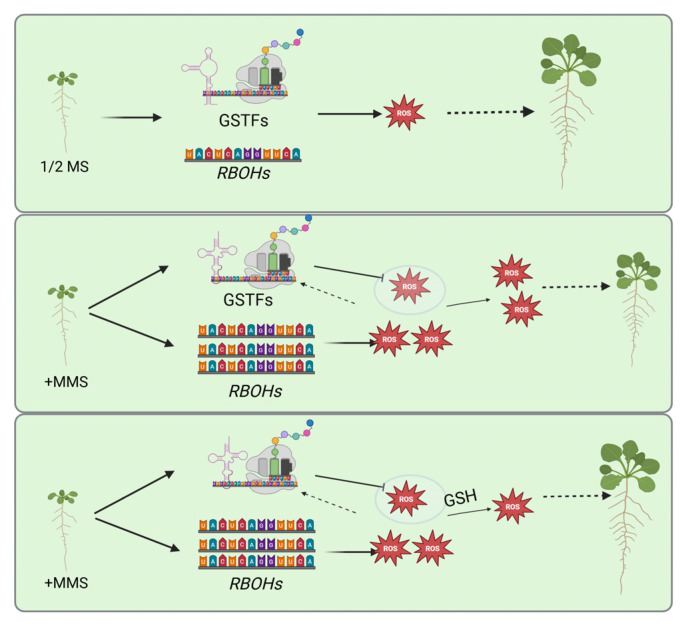
A model for this study. Under normal conditions. ROS levels are maintained at a certain threshold by the plant’s ROS production and removal systems. However, DNA damage agent MMS can alter the transcriptional landscape of *RBOHs*, leading to increased ROS accumulation. Concurrently, plants upregulate the expression of *GSTFs* genes and modify the RNA structure of GSTFs to promote GSTF protein accumulation to protect themselves from the damage of ROS. Despite this, the ROS production is excessive, resulting in inhibited primary root growth. The addition of GSH can remove the excess ROS, thereby rescuing plant growth.

Taken together, the findings of this study suggest that plant redox homeostasis is associated with MMS-induced primary root growth inhibition. The cellular response to DNA damage agent MMS can alter the transcriptional landscape of *RBOHs* genes, leading to overaccumulation of ROS, resulting in inhibited primary root growth. However, to counter the damage of MMS, plants upregulate the expression of genes in the GSTF family and alter the secondary structures of GSTF9 and GSTF10 to remove the ROS. Similarly, the addition of GSH was able to alleviate MMS-induced root growth inhibition, highlighting a novel redox regulation mechanism in MMS response (Figure 6).

These results indicate that the balance between ROS generation and elimination determines the growth status of plants when confronted with MMS. Whereas the mechanism by which MMS affects RNA structure remains an open question. One possibility is that MMS treatment induces the expression of *RBOHs*, leading to ROS accumulation. ROS can directly or indirectly affect the RNA structure in several ways: (1) ROS have also been reported to directly oxidize nucleoside bases (e.g., formation of 8-oxo guanine [53]), which can lead to G-T or G-A transversions if unrepaired, resulting in the mutations in mRNA that ultimately affect RNA structure. (2) Excessive ROS accumulation can lead to DNA break. In the repair, mutations are unavoidable occur [54]. (3) ROS directly modifies the RNA [55]. (4) ROS can act on the enzyme responsible for RNA modification, such as RlmN [56]. Another possibility is that methyl methanesulfonate regulates RNA structure by directly affecting the modification of plant DNA or RNA.

### Limitation for the Study

Here we established a DMS-MaPseq method for the quantitative analysis of in vivo changes in RNA secondary structures after the application of DNA damage agents MMS and revealed the connection between RNA structure and translation. However, there are several caveats and limitations to consider. First, high-quality RNA structure prediction requires deeper sequencing. Second, gene bias in DMS-MaPseq must be taken into account as it can be influenced by RNA expression levels. Third, integrating multi-omics data, such as splicing and translation information, is essential for predicting how changes in RNA structure impact its function. Additionally, designing an experiment to validate the relationship between RNA structure and its function remains a challenging endeavor. Should discuss the results and how they can be interpreted from the perspective of previous studies and of the working hypotheses. The findings and their implications should be discussed in the broadest context possible. Future research directions may also be highlighted.

## 4. Materials and Methods

### 4.1. The Plant Materials and Growth Conditions

*Arabidopsis* Columbia-0 (Col-0) was used in this study. Seeds of *rbohD* (AT5G47910)/*rbohF*(AT1G64060) (CS9555) mutants were obtained from the Arabidopsis Biological Resource Center (Ohio State University, Columbus, OH, USA). The seeds were sterilized by immersing them in a 10% sodium hypochlorite solution and then grown on half-strength Murashige and Skoog agar plates in a growth chamber (Conviron, Canada) under a 16 h photoperiod, 75% humidity, and a temperature of 22 °C.

### 4.2. Primary Root Growth Inhibition Assays

Sterilized seeds were permitted to grow on half-strength Murashige and Skoog agar plates for 5 days, then the seedlings were treated with 2 mL 2 × 10^−3^ g/L MMS in liquid half-strength Murashige and Skoog medium in 24-well micro titre plates. The primary root length was calculated 3 days after 2 × 10^−3^ g/L MMS treatment. For analysis of the effects of GSH on MMS-induced primary root growth inhibition, the 5-day old seedlings were treated with DMSO, MMS, 300 mM GSH, or 300 mM GSH+ MMS for 3 days. Then, the primary root length was calculated. Each cell was applied to 5 seedlings, and each treatment has 3 replicates.

### 4.3. DMS-MaPseq Library Construction

Ten-day old Arabidopsis seedlings growing on the Murashige and Skoog (MS) agar medium were subjected to liquid 1/2 MS in 12-well plates for one day and then treated with 2 × 10^−3^ g/L MMS for 3 days. DMS-MaPseq library construction was followed by the protocol described [57]. Briefly, seedlings with and without MMS treatment were immersed in DMS reaction solution (2% DMS, 40 mM HEPES pH 7.5, 100 mM KCl, and 0.5 mM MgCl_2_) for 20 min. For a negative control, add the same volume of water into the solution. DMS reaction was quenched by addition of β-mercaptoethanol (final concentration 20%, *v*/*v*). Then the samples were collected after washing with distilled water three times. Total RNA was isolated using Trizol according to the manufacturer’s instructions (Invitrogen, Thermo Fisher Scientific, CA, USA). mRNA was purified from total RNA using poly-T oligo-attached magnetic beads. Sequencing libraries were generated using the NEBNext Ultra RNA Library Prep Kit for Illumina (NEB, MA, USA, Catalog#: E7530L). The qualified libraries were pooled and sequenced on Illumina platforms with the PE150 strategy at Novogene Bioinformatics Technology Co., Ltd. (Beijing, China).

### 4.4. DMS Data Analysis

First, we filtered reads using Trimmomatic version 0.39 [58] to trim Illumina adaptors and remove low quality base pairs. In addition, we removed the first two bases as suggested by Wang et al. [57]. We then mapped clean paired-end reads to the reference transcriptome of Arabidopsis thaliana TAIR10 cDNA (https://ftp.ebi.ac.uk/ensemblgenomes/pub/release-58/plants/fasta/arabidopsis_thaliana/cdna/, last accessed on 6 November 2023) using Bowtie2 version 2.5.1 [59] as suggested [60]. We then kept the uniquely mapped reads for downstream analysis. For transcriptome analysis, the FPKM (fragments per kilobase of transcript per million mapped reads) values for known gene models were identified by featureCounts software, followed by Deseq2 to obtain the differential expression genes (DEGs). The threshold of the q-value was determined using the false discovery rate (FDR). In this study, differentially expressed genes (DEGs) were identified with the rules FDR ≤ 0.05 and fold change ≥2. Gene Ontology (GO) enrichment analysis for biological processes was performed, and the results were visualized with the OmicShare tool, an online platform for data analysis (https://www.omicshare.com/tools/home/report/goenrich.html) [61].

For DMS-MaPseq, we followed the workflow of the RNA framework (Figure S5) [62]. We used rf-count in the RNA framework version 2.8.6 to calculate the number of mutations. Raw counts of mutations were then normalized based on the perspective control sample using the scoring method as described [63] and the boxplot normalization method [60]. We used rf-combine to merge transcripts from three replicates with a minimum number of covered bases = 0.1 (—min-values = 0.1). Lastly, we used rf-fold to predict the RNA structure and visualized RNA structures using RNArtist (https://github.com/fjossinet/RNArtist). Details for our pipeline can be found on Github (https://github.com/lyy005/DMS_MaPseq_pipeline).

### 4.5. RNA Isolation and qRT–PCR

Samples treated with MMS were described as above. Total RNA was isolated using Trizol according to the manufacturer’s instructions. First-strand complementary DNA was synthesized using the HiScript II 1st Strand cDNA Synthesis Kit (+gDNA wiper) (Vazyme, Nanjing, China). To assess the quality of cDNA, RT-PCR was conducted with a pair of primers spanning an intron of *ACTIN* (AT5G09810). SYBR Green master mix (Transgen Biotech, Beijing, China) was used for qRT–PCR. The relative expression levels of genes were calculated using the 2(∆Ct) method with *ELONGATION FACTOR1a* (*EF1a*, AT1G07940) used as an internal control. All primers used for qRT–PCR are listed in Supplemental Table S4.

### 4.6. RNA Translation Efficiency Detection

Translation efficiency assays were performed as described previously [64]. Briefly, 0.2 g of Arabidopsis seedlings was used for polysome extraction with an extraction buffer (200 mM Tris-HCl, pH 9.0, 35 mM MgCl_2_, 200 mM KCl, 25 mM EGTA, 1% Triton X-100 (*v*/*v*), 1% IGEPAL CA-630 (*v*/*v*), 5 mM DTT, 50 μg mL^−1^ chloramphenicol, 1 mM phenylmethylsulfonyl fluoride (PMSF), 100 μg mL^−1^ cycloheximide) and placed at 4 °C with shaking for 20 min, then centrifuged at 16,000× *g* for 20 min at 4 °C for twice. Then, 100 mL of supernatant was collected for total RNA extraction. 1.5 mL supernatant was transferred into 15 mL of sucrose buffer (1.75 M sucrose, 400 mM Tris-HCl, pH 9.0, 35 mM MgCl_2_, 5 mM EGTA, 200 mM KCl, 5 mM DTT, 50 μg mL^−1^ chloramphenicol and 50 μg mL^−1^ cycloheximide) and allowed to centrifuge at 200,000× *g* for 4 h at 4 °C and the polysomes were collected by adding 100 µL of DEPC-treated water at the base. Trizol reagent was used to extract total RNAs and polysomal RNA for qRT-PCR analysis.

### 4.7. Protein Extraction and Immunoblot Analysis

To construct GSTF9-GFP and GSTF10-GFP, the coding sequence region of GSTFs was amplified and cloned into a modified pCAMBIA1305 vector with a GFP tag. *Agrobacterium* suspension with OD_600_ = 0.5 containing the vectors for GSTF9 (AT2G30860) or GSTF10 (AT2G30870) gene fused with GFP in infiltration buffer (10 mM MES pH 5.6, 10 mM MgCl2, 100 µM acetosyringone) was injected into the abaxial surface of *N. benthamiana* leaves with syringes. After incubation for 3 days, the signals were observed under a confocal microscope. Meanwhile, inoculated leaf was cut with a hole punch and incubated with 2 × 10^−3^ g/LMMS for 5 h. Then, the GFP signal was observed by the iBright FL1500 Imaging System. Total protein was extracted with protein extraction buffer (4M Urea, 0.1% NP40, 150 mM NaCl, 50 mM pH 7.5 Tris-HCl), and the protein was separated by SDS-PAGE gel electrophoresis and detected with anti-GFP antibody.

## Data Availability

The data of this study are openly available starting 2 May 2025 at the following website: https://dataview.ncbi.nlm.nih.gov/object/PRJNA1175151?reviewer=hvf30birfrmkr0qgndp4frj78j. The code for data analysis can be accessed https://github.com/lyy005/DMS_MaPseq_pipeline.

## Supplementary Materials

The following supporting information can be downloaded at: https://www.mdpi.com/article/10.3390/plants14050780/s1.
