# Supplementary material for: DNA Damaging Agents Induce RNA Structural and Transcriptional Changes for Genes Associated with Redox Homeostasis in Arabidopsis thaliana
Source: Plants (Basel). 2025 Mar 4;14(5):780. doi: 10.3390/plants14050780 (PMC11901513; doi:10.3390/plants14050780)
Supplement: Supplementary file 1 [file plants-14-00780-s001.zip › plants-3453617-supplementary figures.pdf]

plants

MDPI

Supplementary Figure

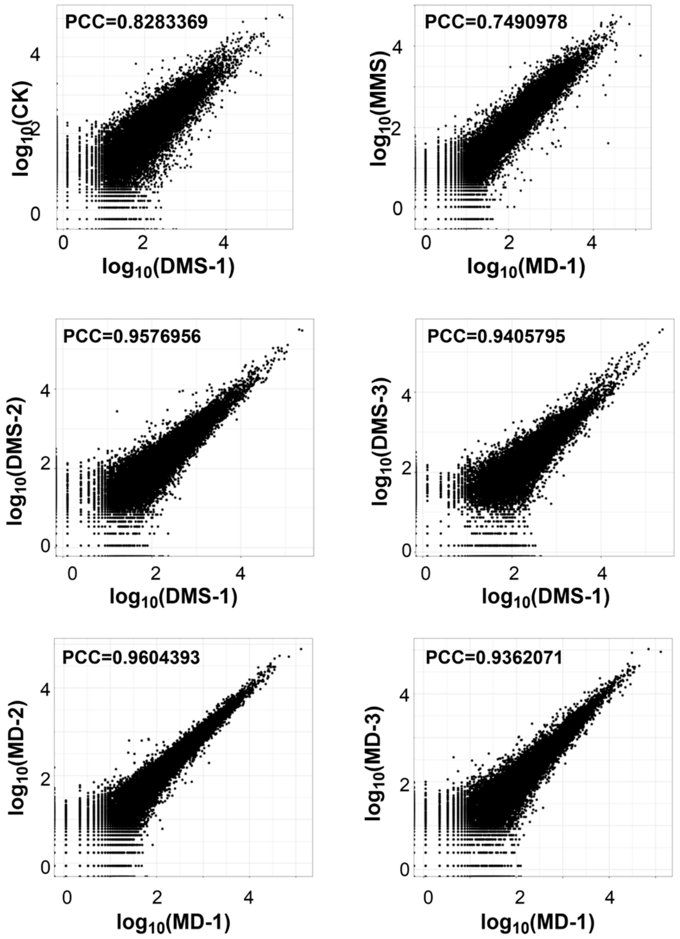

Figure S1. Correlation of mRNA abundance among different groups.

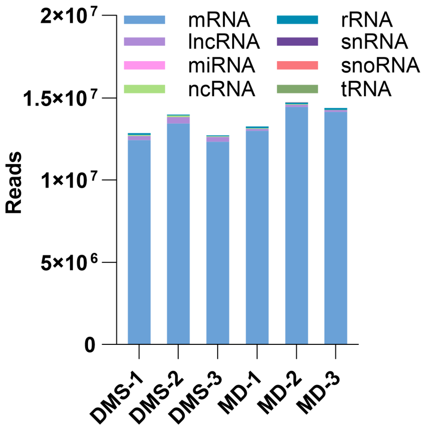

Figure S2. Mapping results of different RNA types among libraries.

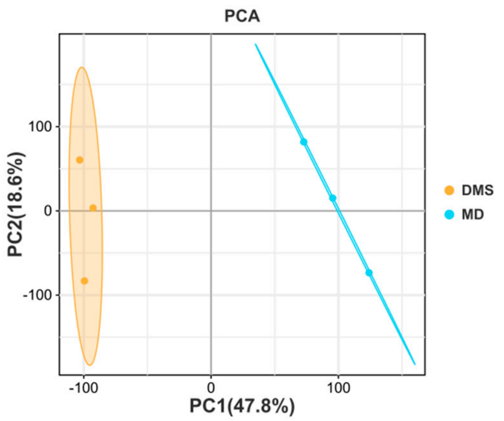

Figure S3. PCA plot for DMS and MD samples.

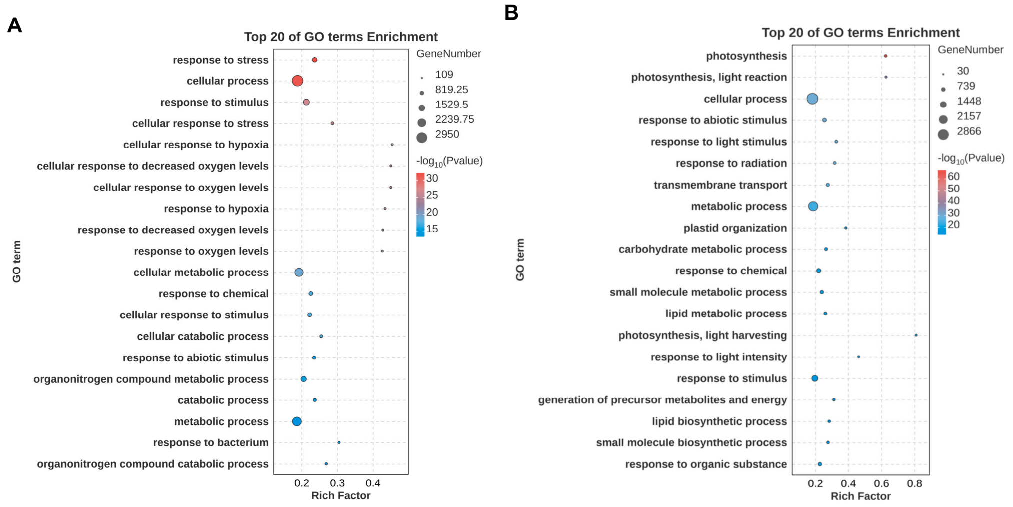

Figure S4. Go analysis for upregualted and downregulated genes. Gene Ontology (GO) enrichment analysis (biological process) for upregulated (A) and downregulated genes was performed and the results visualized with the OmicShare tool, an online platform for data analysis (https://www.omicshare.com/tools/home/report/goenrich.html).

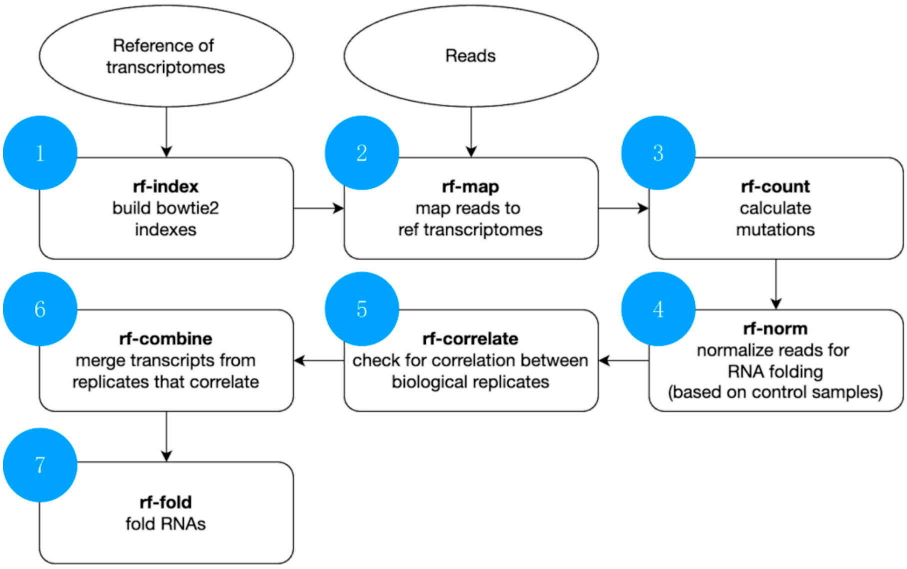

Figure S5. Workflow for DMS-Maseq analysis. (1) Index construction. A Bowtie reference index was constructed with rf-index. (2) Mapping. The paired-end data was mapped to reference genome (Arabidopsis Tair10) with rf-map. (3) Mutation analysis. rf-count in RNA framework version 2.8.6 was used to calculating the number of mutations. (4) Normalization of the data. Raw counts of mutations were then normalized based on the perspective control sample with rf-norm. (5) Samples correlation analysis. rf- correlate allows calculating pairwise correlations of structure probing experiments. (6)We used rf-combine to merge transcripts from three replicates with minimum number of covered bases = 0.1 (--min-values = 0.1). (7) RNA sturctural prediction. rf-fold was used to predict the RNA structure and RNA structures were visualized using RNArtist (https://github.com/fjossinet/RNArtist). The software documentation is available for online access (https://rnaframework-docs.readthedocs.io/en/latest/).

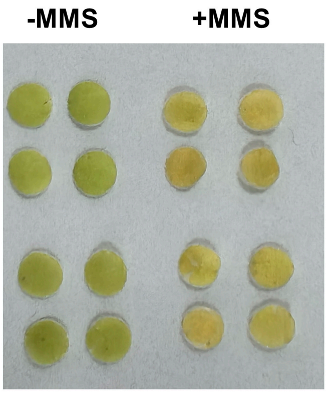

Figure S6. MMS treatment promotes leaf senescence. Leaf disks from the tobacco leaf were treated with or without MMS in water for 7 days.

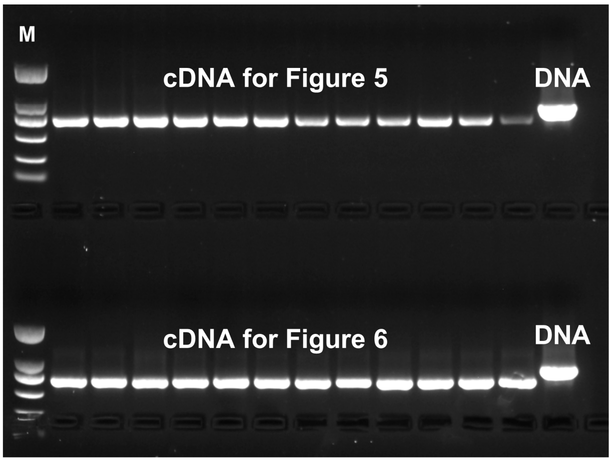

Figure S7. Assessment of DNA contamination in samples. cDNA was obtained by HiScript II 1st Strand cDNA Synthesis Kit (+gDNA wiper). The primers spanning an intron for *ACTIN* was used to verify DNA contamination in samples.

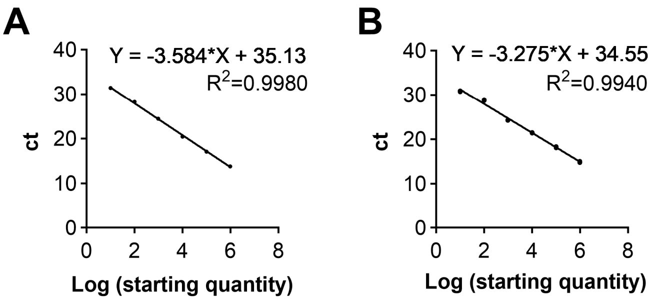

Figure S8. Amplification efficiency curves for the primers of *GSTF9* and *GSTF10* genes. The amplification efficiency values for the primers of *GSTF9* and *GSTF10* genes are 90% and 101%, respectively.

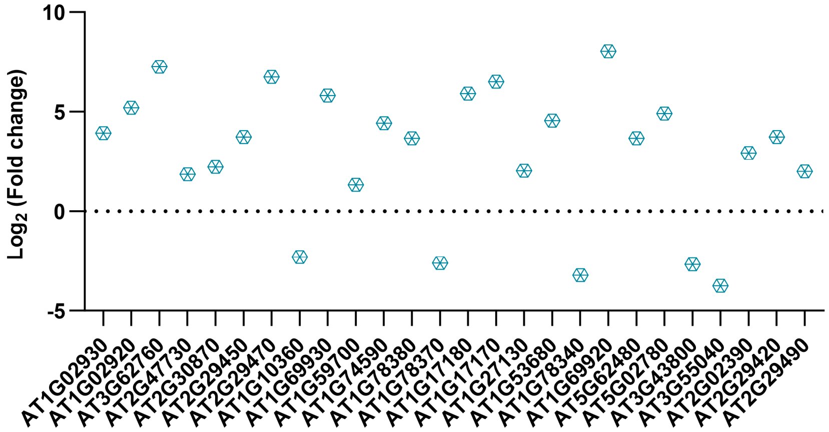

Figure S9. Fold changes of GSTF family genes after MMS treatment from RNA-seq data.
